# Renal Cyst Formation in Fh1-Deficient Mice Is Independent of the Hif/Phd Pathway: Roles for Fumarate in KEAP1 Succination and Nrf2 Signaling

**DOI:** 10.1016/j.ccr.2011.09.006

**Published:** 2011-10-18

**Authors:** Julie Adam, Emine Hatipoglu, Linda O'Flaherty, Nicola Ternette, Natasha Sahgal, Helen Lockstone, Dilair Baban, Emma Nye, Gordon W. Stamp, Kathryn Wolhuter, Marcus Stevens, Roman Fischer, Peter Carmeliet, Patrick H. Maxwell, Chris W. Pugh, Norma Frizzell, Tomoyoshi Soga, Benedikt M. Kessler, Mona El-Bahrawy, Peter J. Ratcliffe, Patrick J. Pollard

**Affiliations:** 1 Nuffield Department of Medicine, Henry Wellcome Building for Molecular Physiology, University of Oxford, Oxford OX3 7BN, UK; 2 Bioinformatics and Statistical Genetics, Wellcome Trust Centre for Human Genetics, University of Oxford, Oxford OX3 7BN, UK; 3 High Throughput Genomics, Wellcome Trust Centre for Human Genetics, University of Oxford, Oxford OX3 7BN, UK; 4 Experimental Histopathology Laboratory, Cancer Research UK London Research Institute, London WC2A 3LY, UK; 5 Department of Histopathology, Royal Marsden Hospital, London WC2A 3LY, UK; 6 Laboratory of Angiogenesis and Neurovascular Link, Vesalius Research Center, VIB Leuven B-3000, Belgium; 7 Laboratory of Angiogenesis and Neurovascular Link, Vesalius Research Center, K.U. Leuven, Leuven B-3000, Belgium; 8 Division of Medicine, University College London, London WC1E 6JF, UK; 9 Department of Exercise Science, School of Public Health, University of South Carolina, Columbia, SC 29208, USA; 10 Institute for Advanced Biosciences, Keio University, 403-1 Daihoji, Tsuruoka, Yamagata 997-0017, Japan; 11 Department of Histopathology, Imperial College, Hammersmith Hospital, London W12 0NN, UK

## Abstract

The Krebs cycle enzyme fumarate hydratase (*FH*) is a human tumor suppressor whose inactivation is associated with the development of leiomyomata, renal cysts, and tumors. It has been proposed that activation of hypoxia inducible factor (HIF) by fumarate-mediated inhibition of HIF prolyl hydroxylases drives oncogenesis. Using a mouse model, we provide genetic evidence that Fh1-associated cyst formation is Hif independent, as is striking upregulation of antioxidant signaling pathways revealed by gene expression profiling. Mechanistic analysis revealed that fumarate modifies cysteine residues within the Kelch-like ECH-associated protein 1 (KEAP1), abrogating its ability to repress the Nuclear factor (erythroid-derived 2)-like 2 (Nrf2)-mediated antioxidant response pathway, suggesting a role for Nrf2 dysregulation in FH-associated cysts and tumors.

## Highlights

► Fh1-associated renal cyst formation is independent of Hif/Phd pathway ► Nrf2-mediated antioxidant response pathway is upregulated following Fh1 loss ► Fumarate modifies cysteine residues in Keap1 by succination

## Significance

**Activation of hypoxia pathways is strongly associated with poor prognosis in cancer. Inactivation of the tumor suppressor gene encoding fumarate hydratase (FH) causes activation of hypoxia signaling, and it has been proposed that this plays a causal role in renal cyst and tumor development. Analyses of mice defective in both FH and HIF signaling reveal that inactivation of Hif-1α but not Hif-2α actually exacerbates renal cyst development. A HIF-independent association has been identified between NRF2 pathway activation and FH deficiency, which correlates with fumarate-mediated modification of cysteine residues in KEAP1, the negative regulator of NRF2. Our data introduce NRF2 dysregulation, rather than HIF activation, as a candidate oncogenic pathway in FH-associated disease.**

## Introduction

Hereditary leiomyomatosis and renal cell carcinoma (HLRCC) is an inherited cancer syndrome in which affected individuals are at risk of developing benign cutaneous and uterine leiomyomas, renal cysts, and aggressive collecting duct and Type 2 papillary renal cell carcinomas (pRCC) (Kiuru et al., 2001; Tomlinson et al., 2002; Toro et al., 2003). The early onset of cysts in hereditary renal cancer syndromes including HLRCC and von Hippel-Lindau (VHL) disease (Kaelin, 2008; Lehtonen et al., 2006) together with observation of dysplastic changes in cystic epithelium and solid tumors arising from cyst walls, strongly suggests that cystic change represents an early stage in carcinogenesis. Patients with HLRCC carry heterozygous germline mutations in the gene encoding the Krebs cycle enzyme fumarate hydratase (FH) and tumor formation is associated with loss of heterozygosity at this locus (Tomlinson et al., 2002). Although FH has a central role in cellular energy metabolism, the mechanisms underlying FH-associated tumorigenesis remain to be defined (Frezza et al., 2011).

Much interest has focused on the possibility that upregulation of one or more transcriptional pathways mediated by hypoxia inducible factor (HIF) may underlie the oncogenic process (Ratcliffe, 2007). In normal cells, oxygen dependent prolyl hydroxylation of HIF-alpha (HIF-α) subunits promotes their degradation by the VHL ubiquitin E3 ligase-proteasome pathway (Kaelin, 2008). In FH-deficient cells and tumors, fumarate accumulates to very high levels, and competitively inhibits the 2-oxoglutarate (2OG) dependent dioxygenases that catalyze HIF prolyl hydroxylation, thus mimicking hypoxia and stabilizing the HIF complex (Isaacs et al., 2005; O'Flaherty et al., 2010; Pollard and Ratcliffe, 2009). This process, termed pseudohypoxia, has been proposed to drive tumor development by activation of oncogenic HIF target genes. In favor of this hypothesis, activation of the HIF transcriptional cascade is strongly associated with aggressive behavior and poor prognosis across a wide range of common human cancers and HIF target genes include many with potentially oncogenic actions in angiogenesis, energy metabolism, invasion and dedifferentiation (Kaelin and Ratcliffe, 2008). HIF is strongly upregulated, both in HLRCC-associated human pRCC and in the hyperplastic renal cysts that develop in mice following targeted inactivation of *Fh1* (the murine homolog of FH), and studies of gene expression patterns in these tissues have revealed strong signatures of HIF activation (Ashrafian et al., 2010; Isaacs et al., 2005; Pollard et al., 2005; Pollard et al., 2007). Based on these findings, it has been proposed that pharmacological downregulation of HIF pathways by agents that promote HIF hydroxylation in the face of high fumarate levels might provide an effective treatment for FH-associated neoplasia (Mackenzie et al., 2007; Tennant and Gottlieb, 2010).

It has been shown (Alderson et al., 2006; Frizzell et al., 2011; Nagai et al., 2007) that fumarate, in addition to its role as an allosteric regulator of 2OG-dioxygenase, also modifies cysteine residues in many proteins, forming S-(2-succinyl)-cysteine (2SC). Critically, these modifications have functional consequences as exemplified by inactivation of glyceraldehyde-3-phosphate dehydrogenase (Blatnik et al., 2008a, 2008b). Recently, we have reported that FH-deficient cells and tumors accumulate high levels of 2SC (Bardella et al., 2011). Furthermore, this modification is highly specific and absent in normal tissues and other tumor types and therefore a candidate mechanism for tumorigenesis.

To define the role, if any, of HIF activation in FH-associated neoplasia, we combined inactivation of Fh1 with Hif-1α, Hif-2α, or both Hif-α isoforms, measured the frequency of renal cyst formation in a mouse model recapitulating the cystic phenotype of the human disease, and compared the outcome with that of genetic inactivation of the Hif prolyl hydroxylases (Phds). To extend our analyses and understanding of events underpinning cyst formation following the loss of FH, and to identify potential HIF-independent oncogenic pathways, we compared gene expression patterns in Fh1- and Fh1; Hif-1α-deficient kidneys, where Fh1-associated profiles are not confounded by Hif activation. We provide evidence for an alternative mechanism by which fumarate may activate oncogenic pathways.

## Results

### Role of Hif in Fh1-Associated Renal Cystic Disease

**Figure 1:**
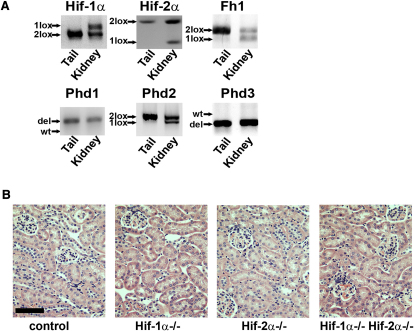
Loss of Hif-α Does Not Initiate Renal Cyst Formation (A) Representative blots of the PCR amplification of genomic DNA from tails and kidneys of mice lacking combinations of *Fh1*, *Hif-1α*, *Hif-2α*, *Phd1*, *Phd2*, and *Phd3* alleles. These show that *Phd1* and *Phd3* are constitutively deleted, whereas null alleles for *Fh1*, *Hif-1α*, *Hif-2α*, and *Phd2* are present only in DNA from the kidney, generated as a consequence of excision of floxed alleles by Ksp-cre in the tubules. (B) H&E staining of kidney sections from control, *Hif-1α^−/−^*, *Hif-2α^−/−^*, and *Hif-1α^−/−^ Hif-2α^−/−^* mice showing that there is no renal cyst development by 40 weeks of age; scale bar = 100 μm.

To assess the role of HIF activation in FH-associated renal cystic disease, we determined if parallel inactivation of Hif-1α or Hif-2α would ameliorate the hyperplastic renal cystic phenotype in mice with renal tubule specific inactivation of *Fh1* (Pollard et al., 2007). Accordingly, mice bearing conditionally inactivated alleles of *Hif-1α* (Cramer et al., 2003; Higgins et al., 2004), *Hif-2α* (Gruber et al., 2007), and *Fh1* (Pollard et al., 2007) were intercrossed with transgenic animals expressing Cre recombinase under the control of a kidney specific cadherin (Ksp) promoter (Shao et al., 2002) to generate mice that were transgenic for Ksp-Cre and homozygous for one or more conditionally inactivated alleles. A total of seven lines were generated as follows: *Fh1^fl/fl^ Ksp-Cre^+/−^* (*Fh1^−/−^*), *Hif-1α^fl/fl^ Ksp-Cre^+/−^* (*Hif-1α^−/−^*), *Hif-2α^fl/fl^ Ksp-Cre^+/−^* (*Hif-2α^−/−^*), *Hif-1α^fl/fl^ Hif-2α^fl/fl^ Ksp-Cre^+/−^* (*Hif-1α^−/−^Hif-2α^−/−^*), *Fh1^fl/fl^ Hif-1α^fl/fl^ Ksp-Cre^+/−^* (*Fh1^−/−^Hif-1α^-^*^/-^), Fh1^fl*/f*^*Hif-2α^fl/fl^ Ksp-Cre^+/−^* (*Fh1^−/−^Hif-2α^−/−^*), and *Fh1^fl/fl^ Hif-1α^fl/fl^ Hif-2α^fl/fl^ Ksp-Cre^+/−^* (*Fh1^−/−^Hif-1α^−/−^Hif-2α^−/−^*). Control animals (*Fh1^+/+^*) were littermates bearing conditional alleles without Ksp-Cre or carrying a wild-type allele with Ksp-Cre. For each allele, PCR amplification of genomic DNA from the kidneys was used to verify efficient tissue-specific recombination (Figure 1A).

**Figure 2:**
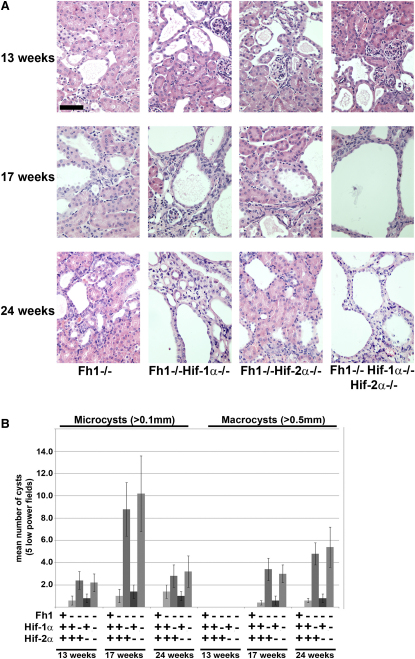
Renal Cyst Formation in Fh1-Deficient Mice Is Independent of the Hif-α Pathway (A) H&E staining of kidney sections from *Fh1^−/−^*, *Fh1^−/−^Hif-1α^−/−^*, *Fh1^−/−^Hif-2α^−/−^*, and *Fh1^−/−^Hif-1α^−/−^ Hif-2α^−/−^* mice at 13, 17, and 24 weeks of age illustrating the development of renal cysts; scale bar = 100 μm. Increased numbers of dilated tubules and microcysts are evident initially, leading to increased size and frequency of cyst formation where Hif-1α is deleted. (B) Analysis of the numbers of microcysts (>0.1 mm) and macrocysts (>0.5 mm) in kidneys from control, *Fh1^−/−^*, *Fh1^−/−^Hif-1α^−/−^*, *Fh1^−/−^Hif-2α^−/−^*, and *Fh1^−/−^Hif-1α^−/−^Hif-2α^−/−^* mice at 13, 17, and 24 weeks of age. Five low-power fields were assessed for cyst numbers from mice in each group (n = 4). Error bars indicate ± 1 SD.

To determine whether loss of the Hif genes alone in kidney tubules would generate major abnormalities that might confound assessment of cyst development in the combined *Fh1^−/−^Hif-α^−/−^* genotypes, we first analyzed kidneys from control, *Hif-1α^−/−^*, *Hif-2α^−/−^*, and *Hif-1α^−/−^Hif-2α^−/−^* mice. No major anatomical abnormalities and in particular no cysts were observed by 40 weeks of age in any of these animals (Figure 1B). By comparison, cyst development in *Fh1^−/−^* mice is observed from 13 weeks of age (Figure 2A) and is followed by ill health or death from renal failure by 50–65 weeks (Pollard et al., 2007). We therefore conclude that inactivation of Hif-1α or Hif-2α, either alone, or in combination, is not sufficient to initiate cyst formation or to disrupt the renal tubule architecture.

Next, we analyzed kidneys from mice in which Hif-1α, Hif-2α, or both had been deleted in renal tubules in parallel with Fh1. Histological analysis was performed at 13, 17, and 24 weeks of age (Figure 2A). Combined deletion of Fh1 and Hif-1α in *Fh1^−/−^Hif-1α^−/−^* mice did not ameliorate the development of cystic disease as had been postulated. In contrast, parallel inactivation of Hif-1α strikingly accelerated both the initiation and progression of cystic disease compared with mice lacking Fh1 alone, evidenced by increased numbers of dilated tubules and microcysts that progressed to larger and more frequent cysts. By comparison, cysts were never observed in control mice (Figures 1B and 2B). Quantification of the numbers of microcysts (>0.1 mm) in kidneys from control, *Fh1^−/−^* and *Fh1^−/−^Hif-1α^−/−^* mice at three time points revealed a significant increase in *Fh1^−/−^Hif-1α^−/−^* mice at 13 and particularly at 17 weeks of age as compared with *Fh1^−/−^* mice (p < 0.01). This marked exacerbation of the cystic phenotype is also seen in the numbers of macrocysts (>0.5 mm) observed at 17 and 24 weeks of age (Figure 2B).

The development of renal cysts in mice deficient for the *Vhl* tumor suppressor gene can be repressed by genetic inactivation of the Hif dimerization partner Hif-1β (Arnt) but not Hif-1α; thus suggesting a causal role for Hif-2α in renal cyst development (Rankin et al., 2006). This is in keeping with a potential oncogenic role for HIF-2α in the pathogenesis of clear cell renal cancer (Kaelin, 2007). Since inactivation of Fh1, like Vhl, could potentially result in stabilization of both Hif-α isoforms, we investigated the role of Hif-2α in cyst development in our model. Combined inactivation of Hif-2α and Fh1 failed to ameliorate either the initiation or development of cysts at the three time points analyzed (Figure 2A) and quantification of microcysts and macrocysts showed no differences from Fh1 deficient mice (Figure 2B). These data suggest that distinct mechanisms underlie Vhl- and Fh1-dependent cyst formation.

Although mice subjected to parallel inactivation of Fh1 and Hif-2α were not phenotypically different from animals in which Fh1 alone was inactivated, an inverse relationship between Hif-1α and Hif-2α activity has been proposed previously in some settings (Carroll and Ashcroft, 2006). Therefore, to assess any compensatory or synergistic effects between the two Hif-α isoforms we inactivated both Hif-1α and Hif-2α in combination with Fh1 in the kidney tubules (Figures 2A and 2B). These mice had an apparently identical phenotype to *Fh1^−/−^Hif-1α^−/−^* mice and therefore, at least in the context of this model, Hif-2α does not appear to have a causal role in the cystic phenotype.

### Cyst Development in Fh1-Deficient Mice Is Independent of Phd Inactivation

**Figure 3:**
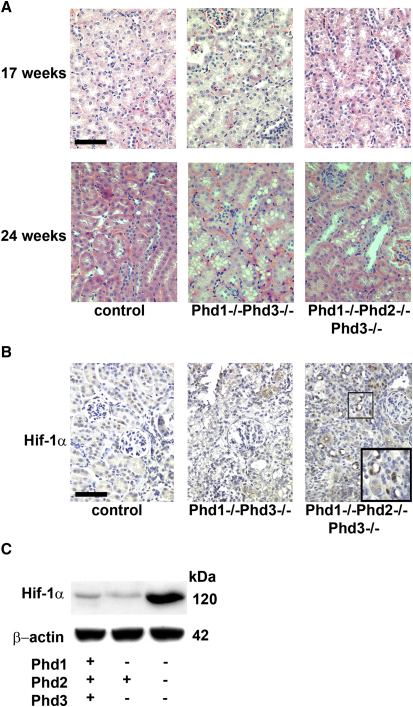
Loss of Prolyl Hydroxylase Domain Enzymes Does Not Initiate Renal Cyst Formation (A) H&E staining of sections of kidneys from wild-type control, *Phd1^−/−^Phd3^−/−^* and *Phd1^−/−^Phd2^−/−^Phd3^−/−^* mice at 17 and 24 weeks of age confirming the absence of renal cysts; scale bar = 100 μm. Kidneys from wild-type, and *Phd1^−/−^Phd3^−/−^* double knockout mice show no abnormal pathology. Kidneys from *Phd1^−/−^Phd2^−/−^Phd3^−/−^* mice have subtle vacuolization, but show no evidence of either tubular dilation or cyst formation. (B) IHC for Hif-1α was performed on kidney sections from 24-week-old control, *Phd1^−/−^Phd3^−/−^* and *Phd1^−/−^Phd2^−/−^Phd3^−/−^* mice. Hif-1α staining (highlighted in insert) is observed only in the *Phd1^−/−^Phd2^−/−^ Phd3^−/−^* mice in the nuclei of cells lining the renal tubules and not in the interstitium; scale bar = 100 μm. (C) Immunoblot of lysates of kidneys from control, *Phd1^−/−^Phd3^−/−^* and *Phd1^−/−^Phd2^−/−^Phd3^−/−^* mice for Hif-1α showing stabilization of Hif-1α in only the kidneys of the *Phd1^−/−^Phd2^−/−^Phd3^−/−^* mice. Protein loading is indicated by β-actin.

HIF prolyl hydroxylation is catalyzed by three closely related enzymes, PHD-1, -2, and -3, (also termed Egln2, -1 and -3) (Epstein et al., 2001). Fumarate, which accumulates in cells lacking FH, mimics hypoxia by allosteric inhibition of the PHDs, allowing HIF to escape destruction and to activate transcription (Isaacs et al., 2005; O'Flaherty et al., 2010). Furthermore, PHDs have been proposed to hydroxylate other substrates in addition to HIF and it is plausible that fumarate-mediated PHD inhibition drives oncogenesis via HIF-independent pathways (Luo et al., 2011; Xie et al., 2009). A number of conditional mouse models exist for the Phd enzymes (Aragonés et al., 2008; Fong and Takeda, 2008; Mazzone et al., 2009). Mice lacking both Phd1 and -3 are viable and develop mild erythrocytosis, although levels of Hif-1α or Hif-2α in the kidney remain normal (Takeda et al., 2008), whereas deletion of Phd2 results in an embryonic lethal phenotype (Takeda et al., 2006). Therefore, to investigate the role of Phds in the regulation of Hif and possibly other relevant pathways affecting cyst formation, we generated mice lacking both Phd1 and −3, (*Phd1^−/−^Phd3^−/−^*) or Phd1, -2, and -3 (*Phd1^−/−^Phd2^fl/fl^ Ksp-Cre^+/−^Phd3^−/−^* from herein termed *Phd1^−/−^Phd2^−/−^Phd3^−/−^*). PCR amplification of genomic DNA from the kidneys verified recombination (Figure 1A). Kidney sections were analyzed from Phd knockout mice at two time points, 17 and 24 weeks of age and compared with littermate controls. Whereas kidneys from control animals had no abnormalities, kidneys from *Phd1^−/−^Phd3^−/−^* double knockout mice and *Phd1^−/−^Phd2^−/−^Phd3^−/−^* triple knockout mice developed subtle tubular cell vacuolization, but no dilation or cyst formation (Figure 3A). Since loss of all three Phds in the kidney failed to induce cyst formation, despite the increased levels of Hif-1α (Figures 3B and 3C), we conclude that cystogenesis in *Fh1*-deficient mice is both Hif and Phd independent.

### The NRF2-Mediated Antioxidant Response Pathway Is Activated in FH-Deficient Cysts and Tumors

**Figure 4:**
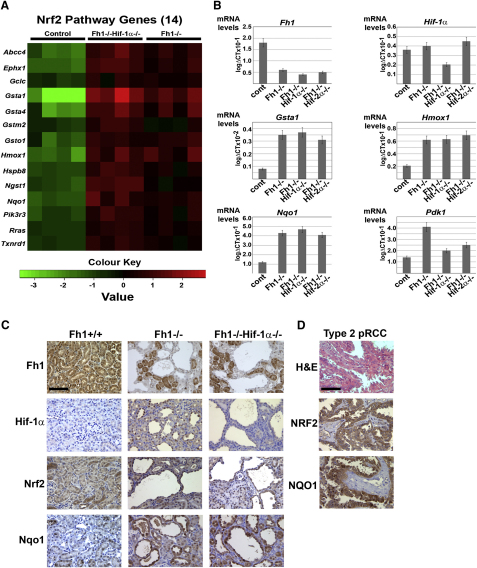
Upregulation of the Nrf2-Mediated Antioxidant Response Pathway in FH-Deficient Cells and Tumors (A) Heat map comparing the patterns of expression for 14 Nrf2 pathway genes for control, *Fh1^−/−^Hif-1α^−/−^*, and *Fh1^−/−^* mouse kidneys (age = 15 weeks, n = 4 per group). Red and green indicate up- or downregulation, respectively. The Heatmap was generated using R (Ihaka and Gentleman, 1996) with differentially regulated genes specific to the Nrf2 pathway. (B) Q-PCR validation of a subset of genes in control, *Fh1^−/−^*, and *Fh1^−/−^Hif-1α^−/−^* mouse kidneys using the same template mRNA as for microarray analysis and compared with renal tissue from *Fh1^−/−^Hif-2α^−/−^* mice confirms significant reduction of mRNA for *Fh1* and *Hif-1α* as expected and upregulation of the Nrf2 target genes *Gsta1*, *Hmox1*, and *Nqo1* in *Fh1^−/−^*, *Fh1^−/−^Hif-1α^−/−^*, and *Fh1^−/−^Hif-2α^−/−^* mouse kidneys. Error bars indicate ± 1 SD calculated from three biological replicates, each assayed in duplicate; p < 0.02 (students t test). Increased expression of the Hif-1α target gene *Pdk1* in *Fh1^−/−^* kidneys is ameliorated by Hif-1α deletion and to a lesser extent by Hif-2α deletion. (C) IHC for Fh1, Hif-1α, Nrf2, and Nqo1 was performed on kidney sections from 17-week-old control, Fh1^−/−^, and Fh1^−/−^ Hif-1α^−/−^ mice. In contrast to the ubiquitous expression of Fh1 in the controls, Fh1 is deleted in cysts of both *Fh1^−/−^* and *Fh1^−/−^Hif-1α^−/−^* mice, but is retained in the interstitium and in a proportion of the renal tubules. Hif-1α is stabilized in the nuclei of cells lining the cysts in *Fh1^−/−^* kidneys, while this staining is absent in the control tissue and *Fh1^−/−^Hif-1α^−/−^* kidneys. Renal cysts from both these groups show increased nuclear expression of Nrf2 and Nqo1 compared with the interstitium and most non-cystic tubules and with the control; scale bar = 100 μm. (D) H&E staining and IHC for NRF2 and NQO1 in pRCC shows strong staining for both in the tumor cells exclusively and not the stroma; scale bar = 100 μm. See also Tables S1 and S2.

To gain a better understanding of the molecular mechanisms causing renal cyst development, comparative genome-wide transcript profiling was performed. Since we observed the largest phenotypic differences in cyst development between control mice and *Fh1^−/−^* or *Fh1^−/−^Hif-1α^−/−^* mice these were chosen as the experimental groups between which we compared gene expression profiles. All mice were aged 15 weeks and therefore in the early stages of cystic disease as determined by histological analysis. Pairwise comparisons between the above groups revealed 489 mRNAs to be differentially regulated (see Table S1available online), which were then subjected to IPA pathway analysis (Ingenuity Systems, http://www.ingenuity.com) as in Table S2. The most significant differentially regulated canonical pathway was the Nrf2 -mediated antioxidant pathway which was upregulated in both *Fh1^−/−^* and *Fh1^−/−^Hif-1α^−/−^* mice (Figure 4A).

To validate the results obtained from the microarray analysis and to quantify the differential expression of some genes in the Nrf2- mediated gene pathway we analyzed a subset of genes by quantitative reverse transcription polymerase chain reaction (Q-PCR) in control, *Fh1^−/−^*, *Fh1^−/−^Hif-1α^−/−^* and *Fh1^−/−^Hif-2α^−/−^* mouse kidneys. First, we quantified the expression of *Fh1* and *Hif-1α* (Figure 4B) and confirmed that *Fh1* is significantly reduced (p < 0.05) in the *Fh1^−/−^*, *Fh1^−/−^Hif-1α^−/−^* and *Fh1^−/−^Hif-2α^−/−^* mouse kidneys compared with controls and that *Hif-1α* was significantly reduced (p < 0.05) in the *Fh1^−/−^Hif-1α^−/−^* mouse kidneys compared with controls, *Fh1^−/−^* and *Fh1^−/−^ Hif-2α^−/−^*. Next, gene expression levels were determined for three of the Nrf2 target genes highlighted in the microarray analysis; glutathione S-transferase alpha 1 (*Gsta1*), heme oxygenase 1 *(Hmox1*), and NAD(P)H dehydrogenase [quinone]1 (*Nqo1*) (Figure 4B). All three transcripts were increased very significantly in *Fh1^−/−^*, *Fh1^−/−^Hif-1α^−/−^* and *Fh1^−/−^Hif-2α^−/−^* mouse kidneys compared with controls (p < 0.02). Also, we confirmed that expression of the Hif target gene pyruvate dehydrogenase kinase, isozyme 1 (*Pdk1*) was increased in *Fh1^−/−^* kidneys, but was reduced by parallel Hif-1α or Hif-2α deletion (Figure 4B). To confirm changes in the expression of Fh1, Hif-1α, Nrf2, and Nqo1 at a cellular level, we undertook immunohistochemistry (IHC) on kidney sections from *Fh1^−/−^* and *Fh1^−/−^Hif-1α^−/−^* mice at 17 weeks of age. IHC staining confirmed that Fh1 (Figure 4C) is deleted in the cysts, but in both groups continues to be expressed in the interstitium and in a proportion of the renal tubules. Hif-1α is stabilized in the nuclei of cells lining the cysts in *Fh1^−/−^* kidneys while this staining is absent in the *Fh1^−/−^Hif-1α^−/−^* kidneys (Figure 4C). Renal cysts from both groups manifest increased nuclear expression of Nrf2; elevated expression in cysts was particularly striking for *Nqo1* as compared with the interstitium and some noncystic tubules (Figure 4C). Similarly, strong staining was observed for NRF2 and NQO1 exclusively in the tumor cells and not the stroma in FH-associated Type 2 pRCC (Figure 4D).

Thus, we conclude that expression of Nrf2, and some Nrf2 target genes, are elevated in those kidney tubule cells that have lost Fh1 and line renal cysts, and that, at least in the mouse model, this occurs independently of Hif-1α and Phds and at an early stage of renal cyst formation. Furthermore, elevated levels of these genes can be detected specifically in the tumor cells of HLRCC-associated Type 2 pRCC.

### Loss of Fh1 Directly Upregulates the NRF2-Mediated Antioxidant Pathway

**Figure 5:**
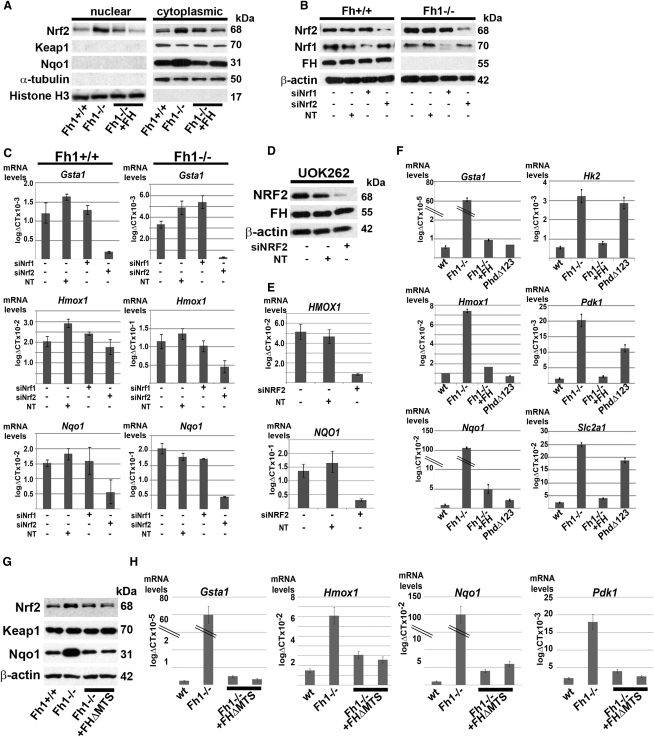
Upregulation of the Antioxidant Pathway in FH-Deficient Cells Is NRF2-Dependent and HIF/PHD-Independent (A) Immunoblot of MEF lysates from *Fh1^+/+^*, *Fh1^−/−^*, and two independent clones of *Fh1^−/−^* reconstituted with wild-type FH (*Fh1^−/−^*+FH) shows increased levels of Nrf2 in both the nuclear and cytosolic fractions of *Fh1^−/−^* cells. Protein levels of *Nqo1* are also increased in *Fh1^−/−^* MEFS. Protein loading for the nuclear and cytoplasmic fractions is indicated by histone H3 and α-tubulin, respectively. (B) Immunoblot of *Fh1^+/+^* and *Fh1^−/−^* MEFs following siRNA knockdown of Nrf1, Nrf2 and a nontargeting (NT) control. Protein loading is indicated by β-actin. (C) Q-PCR analysis following siRNA knockdown of Nrf1 or Nrf2 in *Fh1^+/+^* and *Fh1^−/−^* MEFs. *Gsta1*, *Hmox1*, and *Nqo1* are significantly reduced by depletion of Nrf2 (p < 0.02), but not by either Nrf1 knockdown, or the nontargeting control (NT). (D) Immunoblot of UOK 262 cells for NRF2 and FH following siRNA knockdown. Protein loading is indicated by β-actin. (E) Q-PCR analysis in UOK 262 cells shows a significant reduction of *HMOX1* and *NQO1* expression following siRNA knockdown of NRF2 (p < 0.05), but not in cells treated with a non-targeting (NT) control. (F) Q-PCR analysis of *Gsta1*, *Hmox1*, *Nqo1, Hk2*, *Pdk1*, and *Slc2a1* in *Fh1^+/+^*, *Fh1^−/−^*, *Fh1^−/−^*+FH, and PhdΔ123 MEFs. *Fh1^−/−^* MEFs have significantly elevated levels of antioxidant response- and Hif-target genes, whereas PhdΔ123 MEFs upregulate Hif-target genes, but not antioxidant response genes. (G) Immunoblot of MEF lysate from *Fh1^+/+^*, *Fh1^−/−^* and two independent clones of *Fh1^−/−^* reconstituted with extramitochondrial wild-type FH (*Fh1^−/−^*+FHΔMTS) shows increased levels of Nrf2 and Nqo1 in the *Fh1^−/−^* cells while Keap1 is equivalent in all the lines. Protein loading is indicated by β-actin. (H) Q-PCR analysis of *Gsta1*, *Hmox1*, and *Nqo1* and *Pdk1* in *Fh1^+/+^*, *Fh1^−/−^*, and *Fh1^−/−^*+FHΔMTS MEFs. *Fh1^−/−^* MEFs have significantly elevated levels of antioxidant response and Hif-target genes, which are ameliorated by extramitochondrial FH expression (O'Flaherty et al., 2010). All error bars indicate ± 1 SD calculated from three biological replicates, each assayed in duplicate. See also Figure S1.

The findings of increased expression of Nrf2 and Nrf2 target genes in *FH* mutant material from both hyperplastic cysts of knockout mice and human tumors indicate that the Nrf2-mediated antioxidant response is activated during the development of FH-associated disease. To determine if this occurs as a direct consequence of Fh1 inactivation, rather than indirectly in response to subsequent pathology, we analyzed changes in the expression of Nrf2 target genes and other relevant proteins in vitro in wild-type and *Fh1^−/−^* mouse embryonic fibroblasts (MEFs) and isogenic stable *Fh1^−/−^* transfectants re-expressing human FH (*Fh1^-/^*^-^+FH) (O'Flaherty et al., 2010). In normal cells, the activity of the NRF2 pathway is controlled by KEAP1; a component of an E3 ubiquitin ligase complex that targets NRF2 for degradation (Zhang, 2006). Mutation, deletion, or oxidation of KEAP1 leads to accumulation of nuclear NRF2, enhanced binding to antioxidant response elements (AREs) and activation of downstream target genes (Hayes et al., 2010; Taguchi et al., 2011; Zhang, 2010). To assay the activity of this pathway in MEFs we prepared nuclear and cytoplasmic fractions and analyzed Nrf2, Keap1 and Nqo1 by immunoblotting (Figure 5A). Increased levels of Nrf2 were observed in both the nuclear and cytosolic fractions of *Fh1^−/−^* cells compared with either wild-type cells, or to two independent *Fh1^−/−^*+FH clones. Whereas the level of Keap1 was unaffected by FH status, Nqo1 was clearly increased in *Fh1^−/−^* MEFs relative to the other cell lines.

Since, in addition to NRF2, a complex network of transcription factors, including NRF1, can be recruited to the AREs to modulate transcriptional activity (Biswas and Chan, 2010; Nguyen et al., 2003) we asked whether activation of the antioxidant response observed in Fh1-deficient cells was directly mediated by Nrf2. We therefore depleted Nrf1 or Nrf2 by siRNA in *Fh1^−/−^* or wild-type MEFs. The cells were transfected at 0h and 24h and harvested at 48h for analysis. Efficient and specific knockdown of both Nrf1 and Nrf2 was confirmed at the protein level (Figure 5B). In both cell types, the expression levels of *Gsta1*, *Hmox1*, and *Nqo1* were reduced by depletion of Nrf2, but not by either Nrf1 knockdown, or a non-targeting control (Figure 5C). To determine whether NRF2 also mediates upregulation of the antioxidant pathway in human FH-associated cancer we performed siRNA knockdown of NRF2 in UOK 262 cells, derived from lymph node metastases in an HLRCC patient with aggressive recurring kidney cancer (Yang et al., 2010). Efficient depletion of NRF2 was confirmed by immunoblotting (Figure 5D) and associated with striking reduction of *HMOX1* and *NQO1* (Figure 5E). Taken together, the data indicate that activation of the Nrf2 pathway occurs as a direct consequence of inactivation of FH in mouse and human cells.

### Upregulation of the NRF2-Mediated Antioxidant Pathway Is Independent of HIF Prolyl Hydroxylase Activity

As shown in Figure 4, upregulation of Nrf2 and its target genes *Gsta1*, *Hmox1*, and *Nqo1* were observed in kidneys from *Fh1^−/−^*, *Fh1^−/−^ Hif-1α^−/−^*, and *Fh1^−/−^ Hif-2α^−/−^* mice, suggesting that this occurs independently of Hif. Similarly, Nrf2 and Nqo1 are both increased in cells lining Fh1-associated renal cysts. To further test any relationship to the Hif/Phd pathway we generated PhdΔ123 MEFs that lack all three of the Phd enzymes. Validation of the genotype and confirmation of upregulation of Hif-1α and Hif target genes in these cells is provided in Figure S1. We then compared expression of *Gsta1*, *Hmox1*, and *Nqo1* and the Hif target genes Hexokinase 2 (*Hk2*), *Pdk1* and Glucose transporter 1 (*Slc2a1*) in wild-type, *Fh1^−/−^*, *Fh1^−/−^*+FH, and PhdΔ123 MEFs. Q-PCR analysis (Figure 5F) demonstrated that while *Fh1^−/−^*MEFs express significantly elevated levels of both antioxidant response and Hif-target genes, PhdΔ123 MEFs upregulated Hif-target genes, but not antioxidant response genes. Thus we conclude that upregulation of the antioxidant response pathway in Fh1 deficient cells occurs entirely independently of Hif/Phd dysregulation.

### Upregulation of the Nrf2-Mediated Antioxidant Pathway Is Independent of Mitochondrial Dysfunction

In previous work, we have demonstrated that the intracellular accumulation of fumarate in *Fh1^−/−^* cells can be corrected in the face of a persistent defect in mitochondrial oxidative metabolism by the re-expression of an extramitochondrial form of human FH (*Fh1^−/−^*+FHΔMTS) (O'Flaherty et al., 2010). Therefore, to distinguish whether Nrf2 activation was a consequence of accumulated fumarate or the mitochondrial defect, we compared Keap1, Nrf2, and Nqo1 protein levels between wild-type, knockout, and *Fh1^−/−^*+FHΔMTS MEFs. Only *Fh1^−/−^* MEFs showed elevated levels of Nrf2 and Nqo1, whereas the *Fh1^−/−^*+FHΔMTS MEFs restored the Nrf2 and Nqo1 levels to those of wild-type MEFs (Figure 5G). Similarly, expression of *Gsta1*, *Hmox1*, and *Nqo1* were elevated only in the *Fh1^−/−^* MEFs as compared with wild-type and *Fh1^−/−^*+FHΔMTS MEFs (Figure 5H). Elevation of the Hif target gene *Pdk1* was observed in only the Fh1 knockout MEFs (Figure 5H), consistent with previous work (O'Flaherty et al., 2010). Taken together, these findings suggest that activation of Nrf2 signaling occurs as a direct consequence of fumarate accumulation in *Fh1^−/−^* cells, rather than as a consequence of defective oxidative metabolism.

### Loss of FH Causes Succination of Cysteine Residues of KEAP1 and Abrogation of Its Function to Repress NRF2 Activity

Since the NRF2-KEAP1 pathway is regulated by critical cysteine residues in KEAP1 and proteins containing succinated cysteine residues are detected readily in FH-deficient cells and tumors (Bardella et al., 2011), we postulated that the succination of cysteine residues in Keap1 might account for the increased levels of Nrf2 and its downstream targets observed in Fh1 deficient cells and tissues.

**Figure 6:**
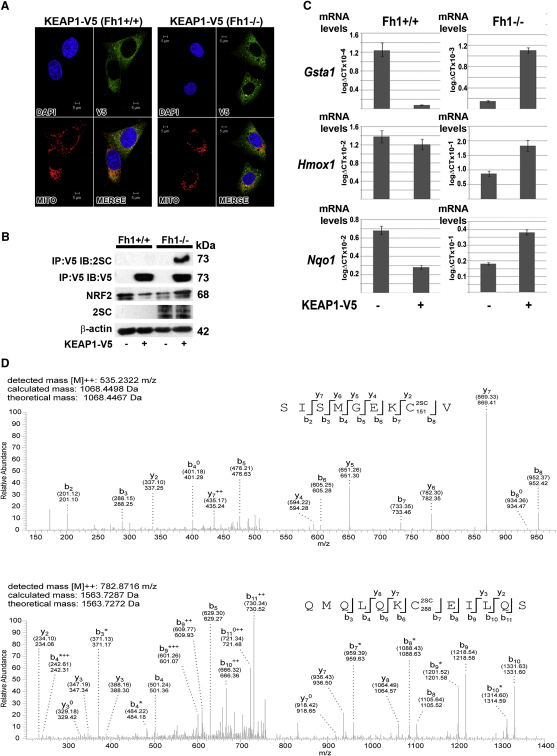
Loss of FH Causes Oxidation of Cysteine Residues of KEAP1 and Abrogation of Its Function to Repress NRF2 Activity (A) Stable transfection and cytoplasmic localization of KEAP1-V5 in *Fh1^+/+^* and *Fh1^−/−^* MEFs was confirmed by IF. Nuclei are stained blue with DAPI, V5 expression indicating KEAP1 cellular localization is labeled green, mitochondria (MITO) are labeled red, and in the last panel the images are merged (MERGE). (B) Immunoblot (IB) analysis of *Fh1^+/+^* and *Fh1^−/−^* MEFs shows that stable expression of KEAP1-V5 reduces levels of Nrf2 in *Fh1^+/+^* MEFs and increases levels of Nrf2 in *Fh1^−/−^* MEFs. Only KEAP1-V5 immunoprecipitated (IP) from *Fh1^−/−^* MEFs exhibits immunoreactivity for 2SC (top panel). Protein levels are indicated by β-actin (bottom panel). (C) Q-PCR analysis of *Gsta1*, *Hmox1*, and *Nqo1* in *Fh1^+/+^* and *Fh1^−/−^* MEFs both with and without stable transfection of KEAP1. Consistent with (B), whereas KEAP1 expression reduces Nrf2 target gene expression in *Fh1^+/+^* MEFS (p < 0.02), stable expression of KEAP1 in *Fh1^−/−^* MEFS increases Nrf2 target gene expression (p < 0.02). Error bars indicate ± 1 SD calculated from three biological replicates, each assayed in duplicate. (D) Succination of human KEAP1 on Cys151 and 288 was identified in *Fh1^−/−^* MEFs transfected with KEAP1 by MS/MS analysis of peptides generated by elastase digestion of KEAP1. MS/MS spectra are shown for peptides SISMGEKCV (corresponding to residues 144–152 of KEAP1) and QMQLQKCEILLQS (corresponding to residues 282–293 of KEAP1), indicating that these are succinated at Cys151 and Cys288, respectively. Both the calculated peptide mass, based on the detected m/z (m: mass, z: charge) value of the doubly charged precursor peptide ion ([M]^2+^), and the theoretical peptide mass, are stated for both peptide spectra. Succination is identified by an additional mass of 116.01 Da added to the corresponding cysteine residue as indicated in the displayed peptide sequence (2SC). Detected N- and C-terminal fragment ions of both peptides are assigned in the spectrum and depicted as follows: b: N-terminal fragment ion; y: C-terminal fragment ion; ^∗^: fragment ion minus NH_3_; ^0^: fragment ion minus H_2_O; and ^2+^: doubly charged fragment ion. Both theoretical mass (in brackets) and detected mass are given for each assigned fragment ion. See also Figure S2.

We were unable to immunoprecipitate endogenous Keap1 with available antibodies; therefore to determine if Keap1 was indeed subject to succination in Fh1 defective cells we generated *Fh1^+/+^* and *Fh1^−/−^* MEFs stably expressing KEAP1-V5 and confirmed cytoplasmic localization of KEAP1 by immunofluorescence (IF) (Figure 6A). To test the function of KEAP1, we analyzed Nrf2 and Nrf2 target gene expression in these cells and demonstrated that as expected, stable expression of KEAP1-V5 reduced levels of Nrf2 protein, and *Gsta1*, *Hmox1*, and *Nqo1* transcript levels in *Fh1^+/+^* MEFs. In contrast, expression of KEAP1-V5 in *Fh1^−/−^* MEFs increased levels of these transcripts indicating that KEAP1 function was strikingly affected by Fh1 status. Furthermore, only KEAP1-V5 immunoprecipitated from *Fh1^−/−^* MEFs exhibited strong immunoreactivity for 2SC (Figure 6B, top panel) implying that at least some cysteine residues within KEAP1 were succinated specifically by high levels of fumarate within *Fh1^−/−^* cells (Figure 6B).

To identify the site(s) of modification in KEAP1 precisely, V5 immunoprecipitates from the KEAP1-V5 transfectants were subject to enzymatic digestion with trypsin, chymotrypsin or elastase and subsequent analysis by tandem mass spectrometry (UPLC-MS/MS). This revealed mass increments of 116.01 Da corresponding precisely to the predicted mass of the succinyl modification on 17 peptides derived from *Fh1^−/−^* but not *Fh1^+/+^* transfectants (Table 1). MS/MS analysis identified succination modification at residues Cys38, Cys151, Cys241, Cys288, Cys319, and Cys613 (Table 1 and Figure 6D). Cys151 and Cys288 have previously been implicated in the regulation of KEAP1 activity by oxidant stress (Nguyen et al., 2009).

Precise quantification of the extent of modification by MS/MS is difficult since the ionization efficiency of modified and unmodified peptide species may differ. However, the addition of two carboxyl residues in the succinated peptide would be predicted to impair ionization, suggesting that succination might be underestimated. The abundance of modified peptides therefore supports the existence of very high levels of succination on KEAP1. Susceptibility of cysteine residues to succination varies, and previous studies have indicated that high pKa thiols such as those found in glutathione (GSH) or N-acetylcysteine are not efficiently targeted for succination (Alderson et al., 2006; Blatnik et al., 2008a). Consistent with this, we observed normal or somewhat elevated levels of reduced GSH in *Fh1^−/−^* kidneys, despite high levels of fumarate (Figure S2), therefore suggesting that fumarate does not react directly with GSH.

**Figure 7:**
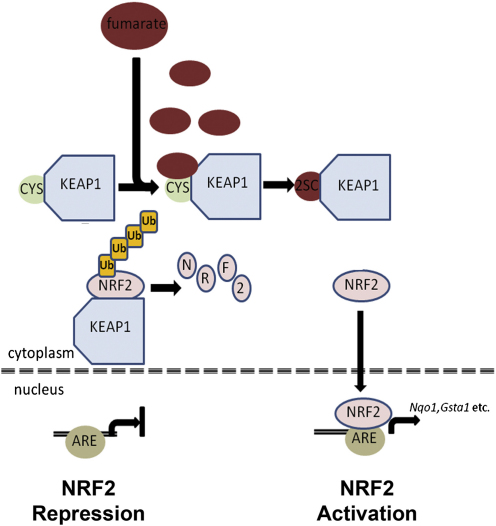
The Potential Roles of Fumarate as an Oncometabolite The KEAP1 protein is part of an E3 ubiquitin ligase, which under normal physiological conditions targets NRF2 for polyubiquitination and subsequent degradation (Zhang, 2006). Critical cysteine residues in KEAP1 are modified by fumarate via succination. We propose that succination impairs the ability of KEAP1 to negatively regulate NRF2, thus facilitating transcription of genes that contain an ARE (Nguyen et al., 2003) in the promoter region.

Taken together, our results suggest that increased levels of fumarate in *Fh1^−/−^* cells promote activation of the Nrf2 antioxidant response pathway by succination of specific redox sensitive cysteine residues in KEAP1 (Figure 7) and not by general oxidant stress.

## Discussion

The unexpected demonstration that the gene encoding the Krebs cycle enzyme fumarate hydratase conforms to the classical genetic model of a tumor suppressor, predisposing individuals carrying germline mutations to cancers bearing somatic inactivation of the second allele (Tomlinson et al., 2002), has raised great interest in defining the associated oncogenic pathway(s).

Structural, biochemical, and biological analyses have established that fumarate, which accumulates in FH-defective cells, binds to PHDs and inhibits their catalytic activity leading to upregulation of HIF transcriptional pathways, as occurs in hypoxia (Hewitson et al., 2007; Koivunen et al., 2007). Hypoxia and activation of the HIF system are commonly associated with aggressive cancer (Harris, 2002), but despite intense investigation, cause and effect have remained difficult to distinguish. Since HIF activation is a direct consequence of inactivation of the FH tumor suppressor, irrespective of hypoxia, this link might indicate causality. Indeed, striking activation of HIF was observed in the mouse model described above and in FH-associated human cancer, as well as in tumors linked to inactivation of the succinate dehydrogenase enzyme complex and mutations in genes encoding isocitrate dehydrogenases 1 and 2, which have also been defined as tumor suppressors or oncogenes (Pollard and Ratcliffe, 2009)).

Our findings clearly demonstrate that despite striking activation of Hif and a number of Hif-target genes in Fh1-deficient cells, neither upregulation of Hif nor inactivation of the Phds is required or responsible for the hyperplastic cystic phenotype observed in the mouse model. Surprisingly we found that, rather than ameliorating cyst development, combined inactivation of Hif-1α (but not Hif-2α) and Fh1, greatly exacerbated cystic hyperplasia. Thus, in this setting, upregulation of Hif-1α appears to exert an antiproliferative effect. While this is apparently at odds with the frequently observed upregulation of HIF-1α in cancer, it is consistent with emerging evidence for differential effects of HIF-1α and HIF-2α in tumor biology. HIF-1α antagonizes MYC function, whereas HIF-2α promotes MYC activity (Gordan et al., 2007), and overexpression of HIF-1α and HIF-2α have contrasting effects on the growth of experimental tumors from VHL-defective RCC lines (Raval et al., 2005). Furthermore, mutational analyses reveal a modest but significant prevalence of HIF-1α inactivating mutations in VHL-associated clear cell RCC (Dalgliesh et al., 2010; Morris et al., 2009; Shen et al., 2011). Nevertheless, the finding that inactivation of Hif-2α had no effect on Fh1-associated cystic disease either alone, or in combination with Hif-1α inactivation, differs from findings reported in a similar mouse model of VHL-associated renal neoplasia. In this latter model, combined inactivation of Arnt, but not Hif-1α, ameliorated Vhl-associated renal cystic disease, implying that Hif-2α might be responsible for the cyst development associated with Vhl loss (Rankin et al., 2006). Hence, we conclude that despite the common activation of HIF pathways in VHL- and FH-associated neoplasia, the oncogenic mechanisms are likely to be different.

Combined inactivation of Fh1 and Hif-1α in our mouse model enabled Fh1 dependent changes in transcript profiles to be interrogated without confounding influences from activation of extensive HIF-dependent transcriptional cascades and revealed striking activation of the Nrf2-mediated antioxidant signaling pathway. Further analyses in cell lines derived from *Fh1^−/−^* MEFs, mouse cystic tissues and FH-associated human cancer demonstrated that activation of the canonical NRF2 antioxidant pathway arose as a direct consequence of FH inactivation. Though we cannot exclude other influences on Nrf2 dysregulation, the demonstration of high levels of succination on critical cysteine residues in KEAP1, the abnormal activity of transfected KEAP1 in *Fh1^−/−^* cells and the maintenance of GSH levels in Fh1^−/−^ cells all argue that Nrf2 activation results from succination of KEAP1 rather than general oxidant stress, at least under the conditions of these experiments (Figure 7). NRF2 acts as a master regulator controlling the ability of mammalian cells to adapt rapidly to stress caused by oxidants and electrophiles, through the induction of ARE containing genes (Nguyen et al., 2003). KEAP1 complexes with Cullin 3 (CUL3) forming an ubiquitin E3 ligase that degrades NRF2. Although not fully understood, the interactions by which KEAP1 controls the levels of NRF2, its cellular localization and transcriptional activity are complex. However, all current models propose that cysteine residues of KEAP1 are modified in response to oxidative stress, resulting in compromised function of the ubiquitin E3 ligase complex that effects proteasomal degradation of NRF2 and enhanced NRF2 stability (Nguyen et al., 2009).

Given that succination of multiple proteins occurs in FH-defective cells and tumors (Bardella et al., 2011), we enquired whether defective KEAP1 function in Fh1 deficient cells might be associated with succination of critical regulatory cysteine residues. MS/MS analysis provided clear evidence of succination on KEAP1 residues Cys151 and Cys288 in association with defective regulation of Nrf2 in *Fh1^−/−^* cells. These two residues are among those cysteines, Cys23, 151, 273, 288, and 613, that are conserved between mouse and human and which have been identified as having functional roles in the activity of Keap1 (Hayes et al., 2010; McMahon et al., 2010; Taguchi et al., 2011). Transgenic complementation studies have shown that Cys273 and 288 are essential for Keap1 to repress Nrf2 activity in vivo, while Cys151 is important in facilitating Nrf2 activation in studies with MEFs from a Keap1 (C151S) transgenic mouse model (Yamamoto et al., 2008). Although Cys613 is part of a zinc sensor system (McMahon et al., 2010) and might be modified by fumarate leading to zinc signaling (Cousins et al., 2006) our microarray data and pathway analyses do not suggest that this pathway is dysregulated in Fh1-associated cystic disease.

Interestingly, a recent study has described activation of Nrf2 by exogenous fumarate both in vitro and in vivo (Linker et al., 2011); similar to our findings, they provide direct evidence that KEAP1 is modified at Cys151, though not at Cys288. Whereas this study utilized cell permeable fumaric acid esters (mono- and dimethylfumarate), we have demonstrated that pathophysiological levels of fumarate associated with cancer are sufficient to succinate KEAP1 and activate Nrf2 signaling.

To our knowledge, mutations of *KEAP1*, *NRF2*, or downstream target genes, which might shed further light on the role of this pathway in FH-associated oncogenesis, have not yet been described in Type 2 pRCC. Given the extensive transcriptional cascade regulated by NRF2, whether and how dysregulation of KEAP1/NRF2 signaling drives oncogenesis requires further investigation. However, both *KEAP1* and *NRF2* somatic missense mutations have been identified in a variety of tumors. Moreover, functional assays and the clustering of mutations at sites that disrupt KEAP1/NRF2 regulation have suggested that dysfunction of KEAP1 contributes in some way to oncogenesis in these settings (Hayes and McMahon, 2009; Taguchi et al., 2011). Though KEAP1/NRF2 dysregulation has been considered as an adaptive response that might particularly affect later stages of oncogenesis, recent data in mouse models of pancreatic and lung cancer, where Nrf2 ablation was associated with reduced cellular proliferation, have suggested an early effect (DeNicola et al., 2011). Our data indicating that Nrf2 dysregulation occurs early in the course of hyperplastic cyst development, as a direct consequence of Fh1 inactivation, are consistent with this possibility.

In summary, our investigations have revealed that despite the striking upregulation of the HIF transcriptional cascade in FH-associated neoplasia, these pathways do not appear to contribute to hyperplastic renal cyst formation, at least in a mouse model that recapitulates many features of the human disease; rather, our findings have raised the possibility of an alternative oncogenic action of fumarate through the activation of antioxidant response pathways by succination of KEAP1, and possibly other proteins with tumor suppressor functions.

## Experimental Procedures

### Mice

All experimental procedures were in line with AACR guidelines and passed ethical review by Oxford University's Medical Sciences divisional Local Ethical Review panel. Experiments were performed under UK Home Office regulations, as required by the terms of the Animal (Scientific Procedures) Act 1986. The *Fh1, Hif-1α*, and *Hif-2α* conditional knockout and *Ksp-cre* mice have all been described previously (Gruber et al., 2007; Higgins et al., 2004; Pollard et al., 2007; Shao et al., 2002) as have the *Phd1*, -*2,* and -*3* knockout mice (Aragonés et al., 2008; Mazzone et al., 2009). Each line had been backcrossed with C57/BL6J for at least five generations and was intercrossed to obtain littermates for the appropriate genotypes. Genotyping was determined by PCR (details on request).

### Microarray Analysis

Total RNA was extracted from each sample using a miVana kit (Ambion). The RNA quantity and quality were determined using the Agilent BioAnalyzer 2100 (Agilent Technologies). Gene expression data were obtained by hybridizing a total of 12 mouse samples from three experimental groups: control, *Fh1^−/−^* and *Fh1^−/−^Hif-1α^−/−^* (n = 4 per group) to Illumina MouseWG-6 BeadChips. Chips were scanned with Illumina BeadArray Reader; GenomeStudioV2010.1 (Illumina Inc) was used for data extraction. Data was imported to GeneSpring GX 11.0.2 (Agilent Technologies, Inc., Santa Clara, CA) normalized with Shift to 75 percentile and baseline transformed to median of all samples to identify significantly differentially expressed genes which were then subject to IPA pathway analysis (Ingenuity Systems, http://www.ingneuity.com).

## Figures and Tables

**Table 1:** Cysteine Residues in KEAP1 Found to Be Succinated in FH^−/−^ MEFs by Tandem Mass Spectrometry

2SC Residue	Peptide Amino Acid Position	Peptide Sequence	Mascot Score	Enzyme
38	34–52	ASTE**C**KAEVTPSQHGNRTF	49	Chymotrypsin
	32–45	MYASTE**C**KAEVTPS	56	Elastase
	35–45	STE**C**KAEVTPS	33	Elastase
151	144–152	SISMGEK**C**V	42	Elastase
	144–155	SISMGEK**C**VLHV	22	Elastase
	145–152	ISMGEK**C**V	13	Elastase
	146–152	SMGEK**C**V	24	Elastase
	146–155	SMGEK**C**VLHV	25	Elastase
241	233–245	SRDDLNVR**C**ESEV	38	Elastase
288	282–293	QMQLQK**C**EILQS	39	Elastase
319	304–320	IFEELTLHKPTQVMP**C**R	47	Trypsin
	304–323	IFEELTLHKPTQVMP**C**RAPK	34	Trypsin
	308–319	LTLHKPTQVMP**C**	23	Elastase
	315–323	QVMP**C**RAPK	25	Elastase
	318–323	P**C**RAPK	15	Elastase
613	602–615	SGVGVAVTMEP**C**RK	51	Trypsin
	607–615	AVTMEP**C**RK	17	Elastase

Sequences for all detected peptide species covering the relevant modified cysteine are shown. The enzyme used to generate the peptide species and the MASCOT score obtained for each peptide is listed accordingly.

## Accession Numbers

Microarray data analysis was deposited in the GEO public database (GSE29988) (http://www.ncbi.nlm.nih.gov/geo/).

## Supplemental Information

Document S1. Two Figures and Supplemental Experimental Procedures
