# Supplementary material for: Renal Cyst Formation in Fh1-Deficient Mice Is Independent of the Hif/Phd Pathway: Roles for Fumarate in KEAP1 Succination and Nrf2 Signaling
Source: Cancer Cell. 2011 Oct 18;20(4):524–37. doi: 10.1016/j.ccr.2011.09.006 (PMC3202623; doi:10.1016/j.ccr.2011.09.006)
Supplement: Document S1. Two Figures and Supplemental Experimental Procedures [file mmc1.pdf]

Cancer Cell, *20*

Supplemental Information

Renal Cyst Formation in Fh1-Deficient Mice Is Independent of the Hif/Phd Pathway: Roles for Fumarate in KEAP1 Succination and Nrf2 Signaling

Julie Adam, Emine Hatipoglu, Linda O'Flaherty, Nicola Ternette, Natasha Sahgal, Helen Lockstone, Dilair Baban, Emma Nye, Gordon W. Stamp, Kathryn Wolhuter, Marcus Stevens, Roman Fischer, Peter Carmeliet, Patrick H. Maxwell, Chris W. Pugh, Norma Frizzell, Tomoyoshi Soga, Benedikt M. Kessler, Mona El-Bahrawy, Peter J. Ratcliffe, and Patrick J. Pollard

Inventory of Supplemental Information

Table S1 is related to Figure 4, provided separately as an Excel file

Table S2 is related to Figure 4, provided separately as an Excel file

Figure S1 is related to Figure 5

Figure S2 is related to Figure 6

Supplemental Experimental Procedures

Table S1, related to Figure 4, provided separately as an Excel file. Differentially expressed genes in kidneys from *Fh1*$^{-/-}$, *Fh1*$^{-/-}$*Hif-1α*$^{-/-}$ and control mice

Gene expression data were obtained by hybridising a total of 12 mouse samples from three experimental groups: control, *Fh1*$^{-/-}$ and *Fh1*$^{-/-}$*Hif-1α*$^{-/-}$ (n=4 per group) to Illumina MouseWG-6 BeadChips. Adjusted p-values below 0.05 were considered significant.

Table S2, related to Figure 4 provided separately as an Excel file. Pathway analysis of microarray data

Gene lists from the microarray experiment were interrogated using IPA pathway analysis (Ingenuity® Systems, www.ingenuity.com). The Nrf2-mediated antioxidant response pathway is the most significantly altered pathway highlighted in comparisons between the three experimental groups, control, *Fh1*$^{-/-}$ and *Fh1*$^{-/-}$*Hif-1α*$^{-/-}$ mice.

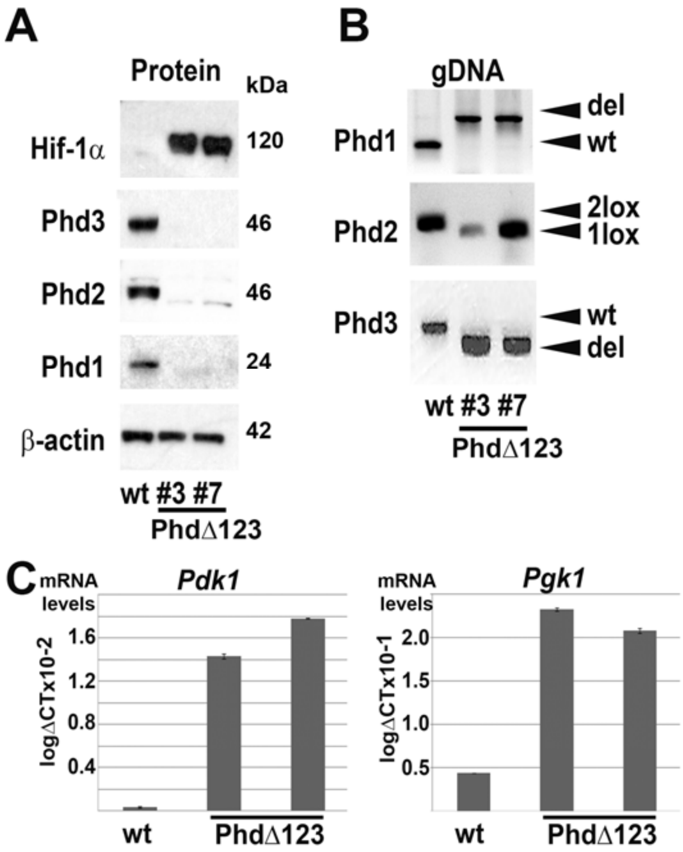

Figure S1, related to Figure 5 - Generation of PhdΔ123 MEFs

(A) Immunoblot of Hif-1α, Phd3, Phd2 and Phd1 in wildtype MEFs and two independent PhdΔ123 MEF clones confirming loss of the three Phd enzymes and upregulation of Hif-1α. Protein loading is indicated by β-actin.

(B) Representative blots of PCR amplification for Phd1, Phd2 and Phd3 from genomic DNA for wildtype MEFs and two independent PhdΔ123 MEF clones confirming deletion of the three Phd enzymes.

(C) Q-PCR analysis confirms upregulation of the Hif target genes *Pdk1* and phosphoglycerate kinase 1 (*Pgk1*) in PhdΔ123 MEFs compared to wildtype MEFs. Error bars indicate ±1 S.D. calculated from three biological replicates, each assayed in duplicate.

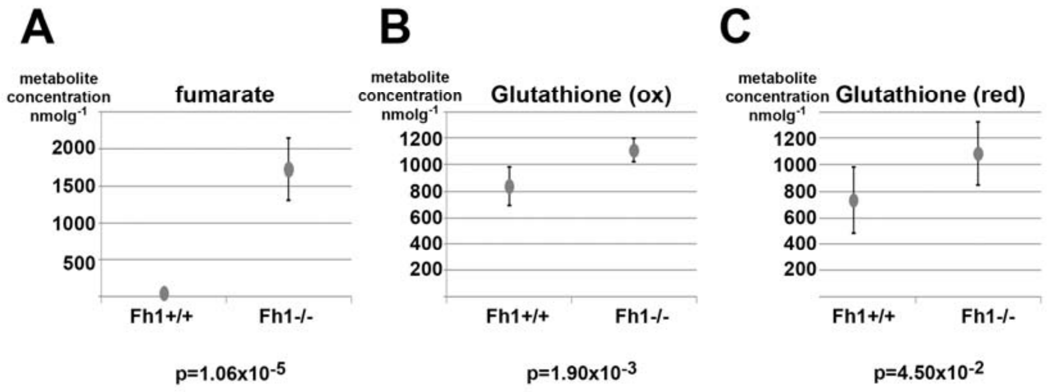

Figure S2, related to Figure 6 - Fumarate and glutathione metabolite analysis in *Fh1*$^{+/+}$ and *Fh1*$^{-/-}$ kidneys

Metabolite analyses were performed by capillary electrophoresis time-of-flight mass spectrometry (CE-TOFMS) on lysates prepared from *Fh1*$^{+/+}$ and *Fh1*$^{-/-}$ kidneys showing that (A) fumarate is significantly higher in the knockout kidneys ($p=1.06 \times 10^{-5}$); while (B) oxidised glutathione ($p=1.90 \times 10^{-3}$); and (C) reduced glutathione ($p=4.50 \times 10^{-2}$) are both slightly elevated in the knockout versus the wildtype kidneys.

Supplemental Experimental Procedures

Generation and maintenance of immortalised and transfected Mouse Embryonic Fibroblasts and HLRCC cancer cell line (UOK 262)

Immortalised *Fh1*$^{-/-}$ and *Phd-1,- 2, -3 null* (PhdΔ1,2,3) MEFs were generated as previously described (O'Flaherty et al., 2010). In brief, MEFs were isolated from littermate embryos and dissected at 14.5 and 12.5 days of gestation respectively using standard protocols. Cells (at passage number 3) were transfected with pBabe-puro SV40 Large T antigen (10^8 PFU) (Gjoerup et al., 2007) and immortalized cells were selected with puromycin. All cells were established and validated by IF, genomic and Q-PCR, and Western blotting. The KEAP1 coding sequence (NM_203500.1) was ligated by PCR into the *Kpn* I and *Not* I sites of pEF1/V5-HisA (Invitrogen) and both *Fh1*$^{+/+}$ and *Fh1*$^{-/-}$ MEFs were transfected with FuGene®6 Transfection Reagent (Roche) following the manufacturer's protocol to generate MEFs with stable KEAP1 expression. All cells were cultured as described previously (O'Flaherty et al., 2010; Sudarshan et al., 2009; Yang et al., 2010).

Cell fractionation

To prepare cytoplasmic and nuclear extracts for immunoblotting, cell pellets were resuspended in hypotonic buffer (20 mM HEPES, pH 8.0, 10 mM KCl, 1 mM MgCl2, 0.1% Triton X-100 and 20% glycerol) and incubated on ice for 10 min. Supernatants collected after centrifugation at 1,500 g for 5 min were used as cytoplasmic fractions. Pellets were washed twice in hypotonic buffer, centrifuged at 5,000 g for 5 min and lysed on ice for 1 h in RIPA buffer (50 mM Tris, pH 7.5, 150 mM NaCl, 0.1% SDS, 0.5% sodium deoxycholate, 1% NP40) followed by centrifugation at 21,000 g for 10 min. All buffers used were supplemented with 'Complete Protease Inhibitor Cocktail' (Roche).

Mass spectrometry (MS) analysis

Proteins were separated by SDS-PAGE and visualized by subsequent Coomassie staining. Gel bands of appropriate size were excised and destained overnight in 50% methanol, 5% acetic acid in water. Proteins were reduced with 10 mM dithiothreitol and then alkylated using 30 mM 4-vinylpyridine for 30 min at 20°C. Proteins were digested with 1 µg elastase, trypsin or chymotrypsin in 50 mM ammonium bicarbonate overnight at 37°C and resulting peptides were extracted from the gel with 50% acetonitrile, 5% acetic acid in water. Resulting peptides were analysed on an Acquity nano UPLC system (Waters) supplemented with a 25 cm C18 column, 1.7 µm particle size (Waters) online coupled to an LTQ Orbitrap Velos (Thermo Scientific). Peptides were eluted by applying a 60 min linear gradient from 5% buffer A (0.1% formic acid in water) to 40% buffer B (0.1% formic acid in acetonitrile) at a flowrate of 250 nl/min (approx. 6000 psi). Full MS scans were performed at a resolution of 30.000. Collision-induced dissociation was performed on the twenty most abundant ions per full MS scan using an isolation width of 1.0 Da. Fragment ions were acquired at a resolution of 7.500. All fragmented precursor ions were actively excluded from repeated MS/MS analysis for 15 s. Raw data was converted to Mascot generic files using msconvert (Kessner et al., 2008) and database searches were performed with MASCOT (Perkins et al., 1999) and CPFP 1.3.0 (Trudgian et al., 2010).

Pathology and Immunohistochemistry

Tissues were fixed in 10% neutral buffered formalin, dehydrated and processed for paraffin wax embedding and sectioning (5µm). Haematoxylin & eosin (H&E) sections were generated for all samples and analysed for routine pathology by two independent pathologists (MEB and GWS). Cyst frequency was determined for each genotype at specified ages in five low power (x10) fields with a minimum of n=5 per group. Immunohistochemistry (IHC) was carried out using the EnVision kit (Dako) as per manufacturer’s protocol as described in (Bardella et al., 2011).

Metabolite analysis (CE-TOFMS)

Frozen samples were homogenized by a cell disrupter (MS-100R; TOMY, Tokyo, Japan) at 2°C, after adding 500 μL of methanol that contained internal standards [20 μM each of methionine sulfone and 2-(N-morpholino)-ethanesulfonic acid (MES)]. The homogenate was then mixed with 200 μl of Milli-Q water and 500 μl of chloroform and centrifuged at 9100 *g* for 4 hr at 4°C. Subsequently, the aqueous solution was centrifugally filtered through a 5-kDa cut-off filter (Millipore) to remove proteins. The filtrate was centrifugally concentrated and dissolved in 50 μL Milli-Q water containing reference compounds (200 μM each of 3-aminopyrrolidine and trimesate). Furthermore, prior to CE-TOFMS analysis, the sample solution for cation and anion was diluted (five times) and (two times) with Milli-Q water, respectively. The concentration of each metabolite was calculated as previously described (Soga et al., 2009).

Immunoblotting and immunoprecipitation

Immunobotting was performed as previously described (Pollard et al., 2007). Immunoprecipitations for V5-tagged KEAP1 were performed using V5-agarose (Sigma) following the manufacturer's protocol.

Immunofluorescence and confocal analysis

Immunofluorescence (IF) and confocal analysis was carried out as previously described (O'Flaherty et al., 2010).

Quantitative reverse transcription PCR

Quantitative reverse transcription PCR analysis was carried out as previously described. (O'Flaherty et al., 2010). Normalization was to β-actin mRNA and relative gene expression was calculated using the ΔΔCT method (Livak and Schmittgen, 2001).

Short interfering RNA (siRNA) knockdown

Cells were transfected with ON-TARGET plus siRNA smartpools (Dharmacon) at a final concentration of 25 nM following the manufacturer's protocol. A non-targeting SMARTpool was included as a control in each assay. Knockdown of protein was determined using immunoblotting and Q-PCR.

Antibodies

The following primary antibodies were used: Hif-1α (Cayman), FH (Autogen Bioclear), 2SC (Nagai et al., 2007), V5 (Invitrogen), R961-25), β-actin (Abcam), Keap1 (Santa Cruz), Nqo1 (Abcam), Nrf1 (Abcam), Nrf2 (Abcam), α-Tubulin (Sigma), and Histone H3 (Abcam). Secondary antibodies were purchased from Dako (HRP-conjugated) and Invitrogen (fluorescent conjugated).

Supplemental References

Gjoerup, O. V., Wu, J., Chandler-Militello, D., Williams, G. L., Zhao, J., Schaffhausen, B., Jat, P. S., and Roberts, T. M. (2007). Surveillance mechanism linking Bub1 loss to the p53 pathway. Proc Natl Acad Sci U S A *104*, 8334-8339.

Kessner, D., Chambers, M., Burke, R., Agus, D., and Mallick, P. (2008). ProteoWizard: open source software for rapid proteomics tools development. Bioinformatics *24*, 2534-2536.

Livak, K. J., and Schmittgen, T. D. (2001). Analysis of relative gene expression data using real-time quantitative PCR and the 2(-Delta Delta C(T)) Method. Methods *25*, 402-408.

Nagai, R., Brock, J. W., Blatnik, M., Baatz, J. E., Bethard, J., Walla, M. D., Thorpe, S. R., Baynes, J. W., and Frizzell, N. (2007). Succination of protein thiols during adipocyte maturation: a biomarker of mitochondrial stress. J Biol Chem *282*, 34219-34228.

Perkins, D. N., Pappin, D. J., Creasy, D. M., and Cottrell, J. S. (1999). Probability-based protein identification by searching sequence databases using mass spectrometry data. Electrophoresis *20*, 3551-3567.

Soga, T., Igarashi, K., Ito, C., Mizobuchi, K., Zimmermann, H. P., and Tomita, M. (2009). Metabolomic profiling of anionic metabolites by capillary electrophoresis mass spectrometry. Anal Chem *81*, 6165-6174.

Sudarshan, S., Sourbier, C., Kong, H. S., Block, K., Valera Romero, V. A., Yang, Y., Galindo, C., Mollapour, M., Scroggins, B., Goode, N.*, et al.* (2009). Fumarate hydratase deficiency in renal cancer induces glycolytic addiction and hypoxia-inducible transcription factor 1alpha stabilization by glucose-dependent generation of reactive oxygen species. Mol Cell Biol *29*, 4080-4090.

Trudgian, D. C., Thomas, B., McGowan, S. J., Kessler, B. M., Salek, M., and Acuto, O. (2010). CPFP: a central proteomics facilities pipeline. Bioinformatics *26*, 1131-1132.

Yang, Y., Valera, V. A., Padilla-Nash, H. M., Sourbier, C., Vocke, C. D., Vira, M. A., Abu-Asab, M. S., Bratslavsky, G., Tsokos, M., Merino, M. J.*, et al.* (2010). UOK 262 cell line, fumarate hydratase deficient (FH-/FH-) hereditary leiomyomatosis renal cell carcinoma: in vitro and in vivo model of an aberrant energy metabolic pathway in human cancer. Cancer Genet Cytogenet *196*, 45-55.
